# Adult human pancreas-derived cells expressing stage-specific embryonic antigen 4 differentiate into Sox9-expressing and Ngn3-expressing pancreatic ducts in vivo

**DOI:** 10.1186/s13287-016-0422-0

**Published:** 2016-11-11

**Authors:** Song Lee, Chan Mi Lee, Song Cheol Kim

**Affiliations:** 1 Asan Institute for Life Science, Asan Medical Center, Seoul, Republic of Korea; 2 Department of Surgery, University of Ulsan College of Medicine, Asan Medical Center, 88 Olympic-ro 43-gil, Songpa-gu, Seoul 05505 South Korea

**Keywords:** Adult human pancreas, Embryonic-like stem cells, Adult stem cells, Pancreatic duct, Stage-specific embryonic antigen 4

## Abstract

**Background:**

Tissue-specific stem/progenitor cells are found in various adult tissues and may have the capacity for lineage-specific differentiation, facilitating applications in autologous transplantation. Stage-specific embryonic antigen 4 (SSEA-4), an early embryonic glycolipid antigen, is expressed in cells derived from adult human pancreas exocrine tissue. Here, we examined the characteristics and lineage-specific differentiation capacity of SSEA-4^+^ cells.

**Methods:**

Human adult partial pancreas tissues were obtained from different donors and cultured in vitro. SSEA-4^+^ and CA19-9^+^ cells were isolated from adult human pancreas exocrine cells using magnetic-activated cell sorting, and gene expression was validated by quantitative polymerase chain reaction. To confirm in-vivo differentiation, SSEA-4^+^ and CA19-9^+^ cells were transplanted into the dorsal subcutaneous region of mice. Finally, morphological features of differentiated areas were confirmed by immunostaining and morphometric analysis.

**Results:**

SSEA-4-expressing cells were detected in isolated pancreas exocrine cells from adult humans. These SSEA-4^+^ cells exhibited coexpression of CA19-9, a marker of pancreatic duct cells, but not amylase expression, as shown by immunostaining and flow cytometry. SSEA-4^+^ cells exhibited higher relative expression of *Oct4*, *Nanog*, *Klf4*, *Sox2*, and *c-Myc* mRNAs than CA19-9^+^ cells. Pancreatic intralobular ducts (PIDs) were generated from SSEA-4^+^ or CA19-9^+^ cells in vivo at 5 weeks after transplantation. However, newly formed PIDs from CA19-9^+^ cells were less abundant and showed an incomplete PID morphology. In contrast, newly formed PIDs from SSEA-4^+^ cells were abundant in the transplanted area and showed a crowded morphology, typical of PIDs. Sox9 and Ngn3, key transcription factors associated with pancreatic development and regeneration, were expressed in PIDs from SSEA-4^+^ cells.

**Conclusions:**

SSEA-4-expressing cells in the adult human pancreas may have the potential for regeneration of the pancreas and may be used as a source of stem/progenitor cells for pancreatic cell lineage-specific differentiation.

**Electronic supplementary material:**

The online version of this article (doi:10.1186/s13287-016-0422-0) contains supplementary material, which is available to authorized users.

## Background

Embryonic stem (ES) cells are undifferentiated cells derived from 5-day preimplantation embryos and are capable of self-renewal, exhibiting pluripotency for the three primary germ layers (ectoderm, endoderm, and mesoderm) [1, 2]. Thus, ES cells are present and have the capacity to differentiate into any cell type only in the embryonic development stage. Cells will then gradually develop into adult cells, and the number of ES/progenitor cells is rapidly reduced, along with a decrease in potential differentiation activity. However, previous studies have suggested that embryonic-like stem cells, also called very small embryonic-like stem cells (VSELs), are present in various adult tissues, such as mammalian ovaries [3, 4], blood [5, 6], and umbilical cord blood [7, 8]. Additionally, some cells expressing ES cell-specific markers, such as Oct4, Nanog, and stage-specific embryonic antigen 4 (SSEA-4), have been identified in cord blood [9] and the adult pancreas [10]. Interestingly, the ES-like cells identified in adult tissue do not have all of the characteristic features of ES cells but still have the ability to differentiate into specific cells. Therefore, many researchers have studied the applications of ES-like cells isolated from adult tissues in regenerative therapies for a variety of diseases [11–14].

The adult pancreas is functionally divided into exocrine and endocrine cells, and the two glands are intimately mixed together into one single organ. Endocrine cells—called islets of Langerhans—release hormones, such as insulin and glucagon, into the bloodstream; these hormones in turn help to control blood glucose levels. Dysfunction of beta cells, which secrete insulin, is directly related to diabetes [15]. Therefore, many researchers have studied beta cell recovery using stem cell therapy. In the adult human pancreas, Afrikanova et al. [10] found that cells positive for SSEA-4, an early embryonic glycolipid antigen, and duct marker proteins could differentiate into pancreatic hormone-expressing cells. They hypothesized that SSEA-4-positive cells may function as human pancreatic stem/progenitors. Unlike other markers of stem cells, such as transcription factors, SSEA-4 is expressed on the surface of ES cells, and it is possible to isolate these cells from tissues using an antigen-antibody reaction-based method without damaging the cells.

In this study, we examined whether isolated SSEA-4-expressing cells from adult human pancreas exocrine cells could differentiate to pancreatic ducts in vivo. We hypothesized that ES cells may remain quiescent for a long period in adult human pancreatic tissue after the end of embryonic development stage and that cells having specific features of embryonic-like stem cells in adult human pancreatic tissue could persist. Therefore, we expected that SSEA-4-expressing cells derived from the human adult pancreas might have the ability to differentiate into pancreatic lineage cells, including pancreatic duct cells, as observed in the early stages of pancreas development.

## Methods

### Human pancreas exocrine cell isolation and culture

Five human adult partial pancreas tissues were obtained from different donors in compliance with the provisions of the Institutional Review Board (IRB) of the Asan Medical Center, Seoul, Korea and in accordance with the Declaration of Helsinki and Good Clinical Practices. To obtain pure exocrine cells, we preferentially removed the islets from the adult pancreas using the modified Ricordi method of pancreas islet isolation [16]. Briefly, collagenase-digested pancreatic tissues were subjected to a continuous-density gradient with Optiprep solution (Sigma-Aldrich, St. Louis, MO, USA) using a COBE 2991 cell processor (Gambro BCT, Inc., Lakewood, CO, USA). Exocrine cells were cultured in adherent in Dulbecco’s modified Eagle medium (DMEM; Gibco, Carlsbad, CA, USA) supplemented with 10 % fetal bovine serum (FBS) and 1 % antibiotics or in suspension in KnockOut DMEM supplemented with 15 % KnockOut serum replacement and 1 % antibiotics in tissue culture plates at 37 °C and 5 % CO_2_. Adherent culture medium and suspension medium were changed at 12 h after the first seeding and then changed every day for 2 days in order to eliminate dying exocrine cells.

### Immunofluorescence staining

For immunocytochemistry, human exocrine cells were cultured on coverslips in 24-well culture plates and fixed with 4 % paraformaldehyde (PFA; Merck, Darmstadt, Germany) for 30 min at 4 °C, followed by washing three times with phosphate-buffered saline (PBS). For detection of cytoplasm markers such as insulin, glucagon, and amylase, cells were permeabilized with 0.3 % Triton X-100 at 4 °C for 5 min and washed three times with PBS. For markers that are expressed in the nucleus, such as Sox9, neurogenin3 (Ngn3), Pdx1, and Ki67, paraffin-embedded slide sections were subjected to antigen retrieval by boiling for 5 min with 10 mM sodium citrate buffer (pH 6.0) and then cooled slowly at room temperature. To block nonspecific binding of the antibody, cell or paraffin slide sections were incubated in 5 % normal goat or normal horse serum for 30 min at room temperature. The primary antibody was incubated with anti-rabbit insulin (1:200; Santa Cruz Biotechnology, Santa Cruz, CA, USA), rabbit anti-human glucagon (1:100; DaKo, Glostrup, Denmark), anti-rabbit amylase (1:200; Sigma-Aldrich), anti-mouse CA19-9 (1:200; Leica Biosystems), and anti-mouse cytokeratin19 (Ck19; 1:200; Santa Cruz Biotechnology) in order to identify endocrine and exocrine cells. Anti-mouse SSEA4 (1:150; Abcam, Cambridge, UK) was used as a marker of embryonic-like stem cells. For detection of pancreatic transcription factors, we used anti-rabbit Sox9 (1:200; Millipore, Temecula, CA, USA), anti-rabbit Ngn3 (1:200; Abcam), and anti-goat Pdx1 (1:10000; Abcam). Proliferation of cells was determined using anti-mouse Ki67 (1:100; Millipore), and differentiation was assessed using anti-goat clusterin (1:200; Santa Cruz Biotechnology). All primary antibodies were incubated overnight at 4 °C. For secondary fluorescence labeling, cells or tissues were incubated with Alexa Fluor 488 goat anti-mouse or anti-rabbit IgG (1:200; Life Technologies, Carlsbad, CA, USA) and Alexa Fluor 594 goat anti-rabbit IgG (1:200; Life Technologies). Hoechst 33342 (1:100; Thermo Scientific, Rockford, IL, USA) or 4′,6-diamidino-2-phenylindole (DAPI, 1:150; Life Technologies) was used to stain nuclei for 3 min at room temperature, and cells were then washed three times with PBS. The slides were visualized under an LSM710 confocal microscope (Carl Zeiss, Oberkochen, Germany).

### Flow cytometry

Adhesion or suspension cultures of exocrine cells were separated with 0.05 % trypsin–EDTA (Gibco) and fixated with 4 % PFA. SSEA-4, CA19-9, and amylase antibodies were conjugated with anti-mouse IgG phycoerythrin (PE) or Alexa Fluor 488 anti-mouse or anti-rabbit IgG (Life Technologies) at RT for 1 h. The exocrine single cells were incubated with PE-conjugated monoclonal anti-SSEA4, Alexa Fluor 488-conjugated anti-CA19-9, or Alexa Fluor 488-conjugated anti-amylase at 4 °C for 30 min in the dark. After washing twice, labeled cells were analyzed on a FACS Caliber flow cytometer (BD Biosciences).

### Cell sorting

The sphere forms of exocrine cells in suspension culture were separated into single cells by treatment with 0.25 % trypsin–EDTA solution (Gibco). After washing twice with PBS, single cells were filtered with a 70-μm nylon cell strainer (Falcon, Corning, NY, USA). For specific sorting of SSEA-4^+^ or CA19-9^+^ cells, 2 × 10^8^ cells were incubated with anti-mouse SSEA-4 (1:150; Abcam) or anti-mouse CA19-9 (1:200; Leica Biosystems) for 30 min at room temperature and then washed twice with PBS. Subsequently, the cells are magnetically labeled with anti-mouse IgG microbeads (Miltenyl Biotec, Auburn, CA, USA). The cell suspensions were then loaded onto a magnetic-activated cell sorting (MACS) column. The magnetically labeled SSEA-4^+^ or CA19-9^+^ cells were isolated by elution with PBS after removing the column from the magnetic field. SSEA-4^–^ or CA19-9^–^ cells were obtained from each nonbinding negative fraction.

### Reverse-transcription polymerase chain reaction

Total RNA was extracted from crude exocrine cells on culture days 2, 4, 6, and 8 or pure fractions of separated cells after MACS sorting using TRIzol (Invitrogen) according to the manufacturer’s instructions. cDNA was synthesized from 0.5–1 μg RNA template using oligo-dT primers with a SuperScript III reverse transcription kit (Invitrogen) at 50 °C for 60 min and 70 °C for 15 min. Polymerase chain reaction (PCR) was performed using rTaq Plus Master Mix (ELPIS Biotech, Seoul, South Korea) with gene-specific forward and reverse primers (Additional file 1: Table S1a). PCR amplification conditions were as follows: initial denaturation at 95 °C for 5 min, followed by 30 cycles at 95 °C for 20 s, 55 °C or 57 °C for 20 s, and extension at 72 °C for 20 s, with a final extension at 72 °C for 3 min. Reverse-transcription-PCR results were normalized against the β-actin housekeeping gene.

### Quantitative PCR

Quantitative PCR (qPCR) products were monitored with a TaqMan probe using AccuPower Plus DualStar qPCR premix (Bioneer, Daejeon, Korea). The TaqMan assays used the 5′ to 3′ exonuclease activity of Taq DNA polymerase, and each reaction contained a gene-specific primer and a fluorescent dye-labeled TaqMan probe. The probe contained the 5′-reporter dye FAM (6-carboxyfluorescein) and the 3′-quencher dye TAMRA (carboxytetramethylrhodamine), and each probe was designed to anneal to the target sequence between the forward and reverse PCR primers. Human ES cells expressing gene-specific primers were also designed (Additional file 1: Table S1b). The qPCR program included a three-step reaction, with predenaturation at 95 °C for 5 min followed by 40 cycles of denaturation at 95 °C for 15 s, annealing at 55 °C or 57 °C for 15 s, and extension/detection at 72 °C for 15 s. After the reaction was completed, gene expression analyses using the 2^−(△△Ct)^ method were performed in order to calculate relative expression.

### In-vivo transplantation

We used 8-week-old male BALB/c nude mice (*n* = 3; Orient Bio Inc., Gyeonggi, Korea) for cell transplantation. All procedures, including the experimental animal treatment and surgical procedures, were approved by the Institutional Animal Care and Use Committee of Asan Medical Center (AMC). Anesthesia was achieved by inhalation of gaseous nitrous oxide–oxygen and isoflurane. SSEA-4-positive, SSEA-4-negative, CA19-9-positive, or CA19-9-negative cells (1 × 10^7^ total cells) were inoculated with Matrigel subcutaneously into the dorsal region in each mouse. After 5 weeks, mice were sacrificed, and transplanted cells were collected, fixed with 4 % PFA, and embedded in paraffin.

### Morphometric analysis

Each of the separated cell populations was transplanted with Matrigel into the dorsal subcutaneous region of the mice, allowing easy sampling. The paraffin-embedded transplanted cells were sectioned to 5-μm thickness and stained with hematoxylin and eosin (H&E). Three to five serial sections were examined for each specimen. To measure the area of newly formed pancreatic ducts on H&E-stained slides, the tissues were photographed with lower magnification (10×) to integrate whole-tissue images using Photoshop (Microsoft Co., San Jose, CA, USA). We delineated the outermost newly formed pancreatic ducts to determine the area of each regenerating lobule relative to the total transplant area. The dimensions of each newly formed pancreatic duct and whole tissues on slides were defined as the total transplanted area and measured area total transplantation using ImageJ (1.47v; Wayne Rasband, NIH, USA).

To measure of numbers of Sox9-positive or Ngn3-positive cells in newly formed pancreatic ducts, nuclear staining was performed with a FITC fluorescent dye and quantified using Image Pro Plus (5.1.0.20v; Media Cybernetics, Inc., Rockville, MD, USA). The number of positively stained cells was determined relative to the total number of pancreatic ductal cells stained with DAPI. Data from three different animals in each group were used for statistical analysis. For standard calculations, the area sizes (mm^2^) and cell counting numbers were converted into percentages (%).

### Statistical analysis

Continuous normally distributed variables are summarized and presented as the mean ± standard deviation (SD). qPCR data were presented as the mean value, determined using duplicates. To calculate the relative expression, the expression of the control was fixed at 1. Statistical analysis was performed using SigmaPlot 8.0 statistical software (SPSS Inc., Chicago, IL, USA), and two-sided Student’s *t* tests were used. Differences with *p* < 0.05 were considered significant.

## Results

### SSEA-4-expressing cells in adult human exocrine pancreas cells

**Fig. 1:**
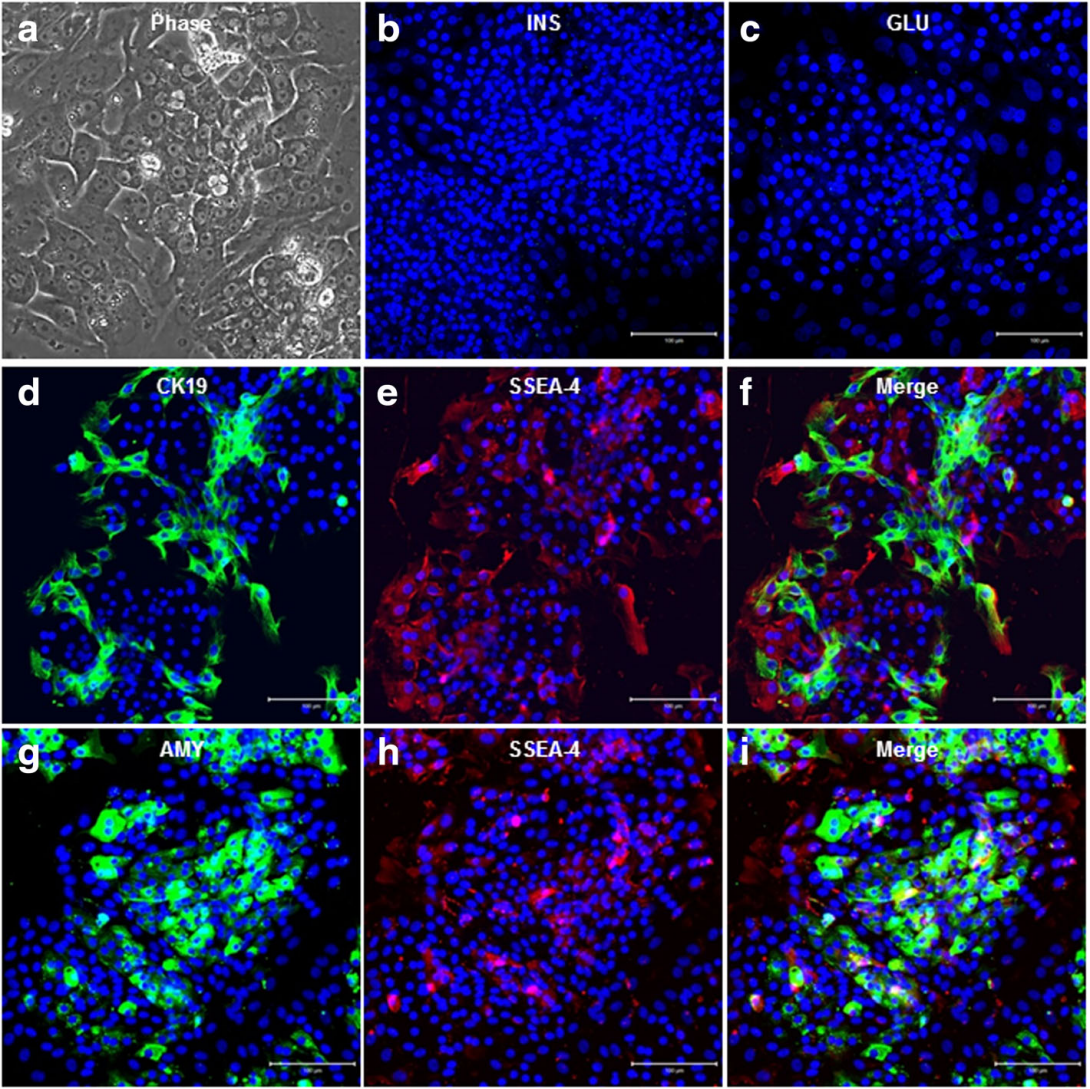
SSEA-4 expression in adult human exocrine cells. **a** Morphology of adult human pancreas exocrine cells during in-vitro adhesion culture. **b** Cultured exocrine cells obtained during human islet isolation. **c** Glucagon expression in adhesion exocrine cell culture. **d** CK19 expression in exocrine cells. CK19 plus SSEA-4 staining is also shown. **e** SSEA-4 expression in exocrine cells. SSEA-4 plus CK19 expression is also shown. **f** Coexpression of SSEA-4 and CK19. **g** Amylase expression in exocrine cells. Amylase plus SSEA-4 staining is also shown. **h** SSEA-4 plus amylase expression. **i** Coexpression of amylase and SSEA-4 in exocrine cells. *Scale bars*: 100 μm. *AMY* amylase, *CK19* cytokeratin 19, *GLU* glucagon, *INS* insulin, *SSEA-4* stage-specific embryonic antigen 4

**Fig. 2:**
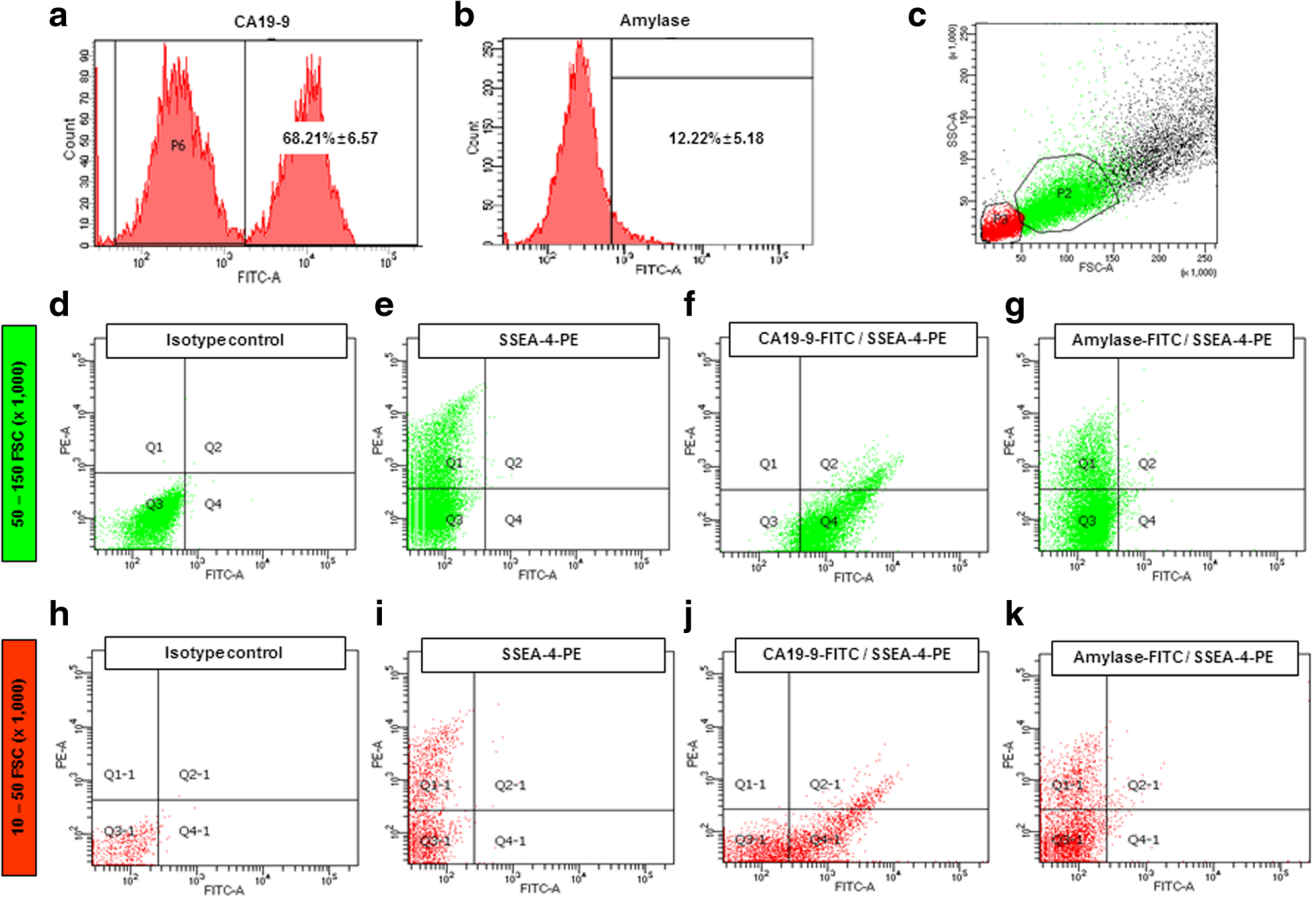
Distribution of SSEA-4-expressing cells in adult human pancreatic exocrine cells. **a** CA19-9 expression in cultured exocrine cells (68.21 ± 6.57 %). **b** Amylase expression in cultured exocrine cells (12.22 ± 5.18 %) **c** Distribution of total cultured exocrine cells plotted according to cell size (common cell size: *P2*; small cell size: *P3*). **d** Negative isotype controls were analyzed using PE-conjugated IgG and FITC-conjugated IgG. **e** Distributions of SSEA-4 expressing cells in exocrine cells (*Q1*: 21.57 ± 3.17 %). **f** Correlation analysis of CA19-9 and SSEA-4 expression in exocrine cells: SSEA-4^+^ cells (*Q1*: 3.02 ± 1.73 %), SSEA-4^+^/CA19-9^+^ cells (*Q2*: 9.65 ± 2.68 %), SSEA-4^–^/CA19-9^–^ cells (*Q3*: 10.92 ± 2.47 %), and SSEA-4^–^/CA19-9^+^ cells (*Q4*: 71.35 ± 11.58 %). **g** Correlation analysis of amylase and SSEA-4 expression in exocrine cells in common cell size gating: SSEA-4^+^ cells (*Q1*: 18.20 ± 3.12 %), SSEA-4^+^/amylase-positive cells (*Q2*: 1.07 ± 0.58 %), SSEA-4^–^/amylase-negative cells (*Q3*: 80.85 ± 6.56 %), and SSEA-4^–^/amylase-positive cells (*Q4*: 10.75 ± 4.235). **h** Negative isotype controls in small cell size gating. **i** Distribution of SSEA-4-expressing cells (*Q1*: 12.44 ± 3.45 %). **j** Correlation analysis of CA19-9 and SSEA-4 expression in small-sized exocrine cells: SSEA-4^+^ cells (*Q1*: 1.97 ± 0.82 %), SSEA-4^+^/CA19-9^+^ cells (*Q2*: 7.72 ± 0.86 %), SSEA-4^–^/CA19-9^–^ cells (*Q3*: 66.20 ± 7.84 %), and SSEA-4^–^/CA19-9^+^ cells (*Q4*: 23.17 ± 25.76 %). **k** Correlation analysis of amylase and SSEA-4 expression in small cell size gating: SSEA-4^+^ cells (*Q1*: 7.32 ± 0.83 %), SSEA-4^+^/amylase-positive cells (*Q2*: 0.67 ± 0.36 %), SSEA-4^–^/amylase-negative cells (*Q3*: 90.07 ± 4.22 %), and SSEA-4^–^/amylase-positive cells (*Q4*: 5.12 % ± 2.59 %). *IgG-PE* phycoerythrin, *SSEA-4* stage-specific embryonic antigen 4

The isolated crude exocrine cells from the adult human pancreas attached properly and proliferated rapidly on tissue culture plates. The morphologies of cultured exocrine cells resembled typical epithelial-like cells (Fig. 1a). These cured exocrine cells mostly consisted of pancreatic duct cells and acinar cells, confirmed by immunocytochemistry with anti-Ck19, a marker of pancreatic duct cells (Fig. 1d), or anti-amylase, a typical digestive juice enzyme secreted by acinar cells (Fig. 1g). However, insulin (Fig. 1b) and glucagon (Fig. 1c) were not found. SSEA-4, a specific marker of ES cells, was also detected in the crude exocrine cells (Fig. 1e, h). In addition, few SSEA-4-expressing cells exhibited coexpression of CA19-9 (Fig. 1f) or amylase (Fig. 1i). We conducted FACS analysis in order to confirm the quantitative distribution of SSEA-4^+^ cells in adult human exocrine cells. In adult human exocrine cells culture, CA19-9^+^ cells were detected in substantial amounts (68.21 ± 6.57 %; Fig. 2a) and amylase-positive cells were detected in moderate amounts (12.22 ± 5.18 %; Fig. 2b). To identify SSEA-4-expressing cells, we analyzed separated cell populations according to cell size (common cell size: 50,000–150,000 FSC; small cell size: 10,000–50,000 FSC; Fig. 2c); this allowed us to confirm whether SSEA-4^+^ cells in adult human exocrine cells belonged to the subtype of VSEL cells, which are known to be present in adult tissues. Expression levels of SSEA-4^+^ cells were similar in cells with a common cell size (21.57 ± 3.17 %; Fig. 2e, Q1) and cells with a small cell size (12.44 ± 3.45 %; Fig. 2i, Q1-1). Cells coexpressing SSEA-4 and CA19-9 were found in 9.65 ± 2.68 % of cells with a common cell size (Fig. 2f, Q2) and in 7.72 ± 0.86 % of cells with a small cell size (Fig. 2j, Q2-1). In addition, coexpression of amylase and SSEA-4 was observed in only 1.07 ± 0.58 % of cells with a common cell size (Fig. 2g, Q2) and in 0.67 ± 0.36 % of cells with a small cell size (Fig. 2k, Q2-1). Based on these results, cells expressing SSEA-4 were relatively rare in adult human exocrine pancreas cells and could not be distinguished based on cell size. Moreover, most SSEA-4^+^ cells were present in pancreatic duct cells.

### Characterization of pure isolated SSEA-4^+^ cells from the adult human exocrine pancreas

Pancreas progenitor cells that can be differentiated into endocrine cells, including insulin-producing cells, were identified in pancreatic duct cells. However, not all pancreatic duct cells are progenitor cells, and many other factors determine the fate of pancreatic progenitor cells. We hypothesized that SSEA-4 may be used as a marker of adult human pancreatic progenitor cells and that SSEA-4^+^ cells may have the capacity for differentiation. Therefore, we analyzed purified SSEA4^+^, SSEA4^–^, CA19-9^+^, and CA19-9^–^ cells from the adult human exocrine pancreas.

**Fig. 3:**
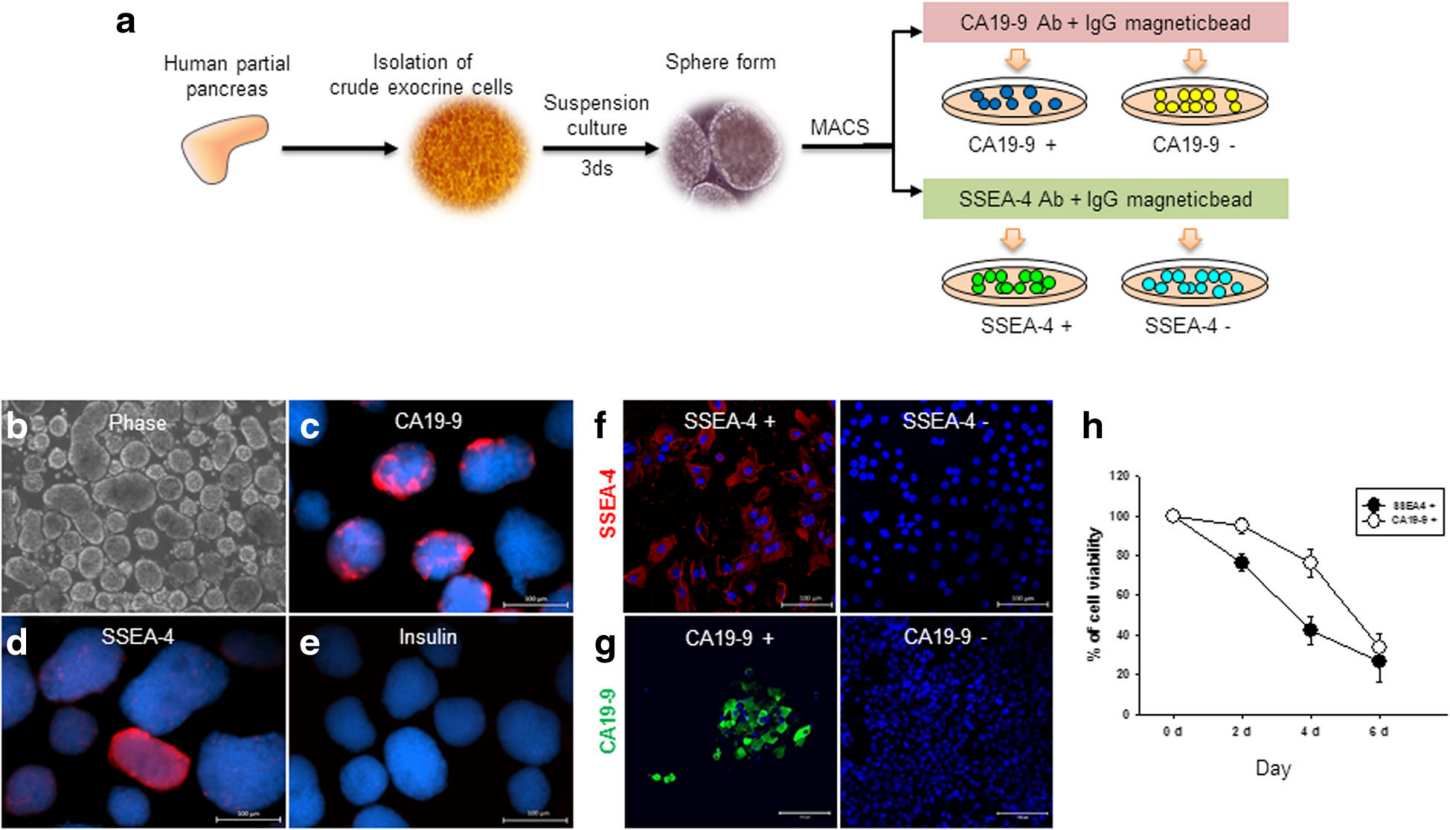
Suspension cultures of adult human pancreas exocrine cells and separation for collection of pure SSEA-4^+^ cells. **a** Processes used for collection of pure single cells. Isolated crude exocrine cells from human partial pancreas tissues formed spheres during suspension culture. Exocrine cell spheres were separated into single cells by TE, and specific positive/negative cells were selected by MACS. **b** Spherical morphology of exocrine cells during suspension culture. **c** CA19-9 expression in spherical exocrine cells. **b** SSEA-4 expression in spherical exocrine cells. **e** Insulin expression in spherical exocrine cells. **f** SSEA-4 expression in cultured SSEA-4^+^ and SSEA-4^–^ cells. **g** CA19-9 expression in cultured CA19-9^+^ and CA19-9^–^ cells. **h** Viability of selected pure single SSEA-4^+^ cells or CA19-9^+^ cells during culture. *Scale bars*: 100 μm. *Ab* antibody, *MACS* magnetic-activated cell sorting, *SSEA-4* stage-specific embryonic antigen 4

In the initial culture of exocrine cells, we evaluated adherent cultures; however, a substantial number of cells could not reattach to the plates during passaging. Therefore, exocrine cells were cultured in suspensions immediately after isolation from the adult human pancreas in order to allow for continuous culture. In 3-day suspension cultures, exocrine cells aggregated and formed spheres (Fig. 3a, b). CA19-9-expressing pancreatic duct cells (Fig. 3c) and SSEA-4-expressing cells (Fig. 3d) were detected consistently; however, insulin-expressing cells were not detected (Fig. 3e) in sphere exocrine cells. Separate preparations of pure pancreatic duct cells and SSEA-4^+^ cells were collected using MACS with anti-CA19-9 or anti-SSEA-4 antibodies, respectively (Fig. 3a). In pure cell culture after separation, we confirmed that SSEA-4 was expressed only in SSEA-4^+^ cells and not in SSEA-4^–^ cells (Fig. 3f), whereas CA19-9 was expressed only in CA19-9^+^ cells (Fig. 3g), as determined by immunocytochemistry. The purified cells, however, exhibited decreased cell viability for both SSEA-4^+^ and CA19-9^+^ cells during culture for 6 days (Fig. 3h). Based on these results, isolated pure single cells appeared to have features of primary cells and were therefore not able to grow when cultured as single cells in vitro.

**Fig. 4:**
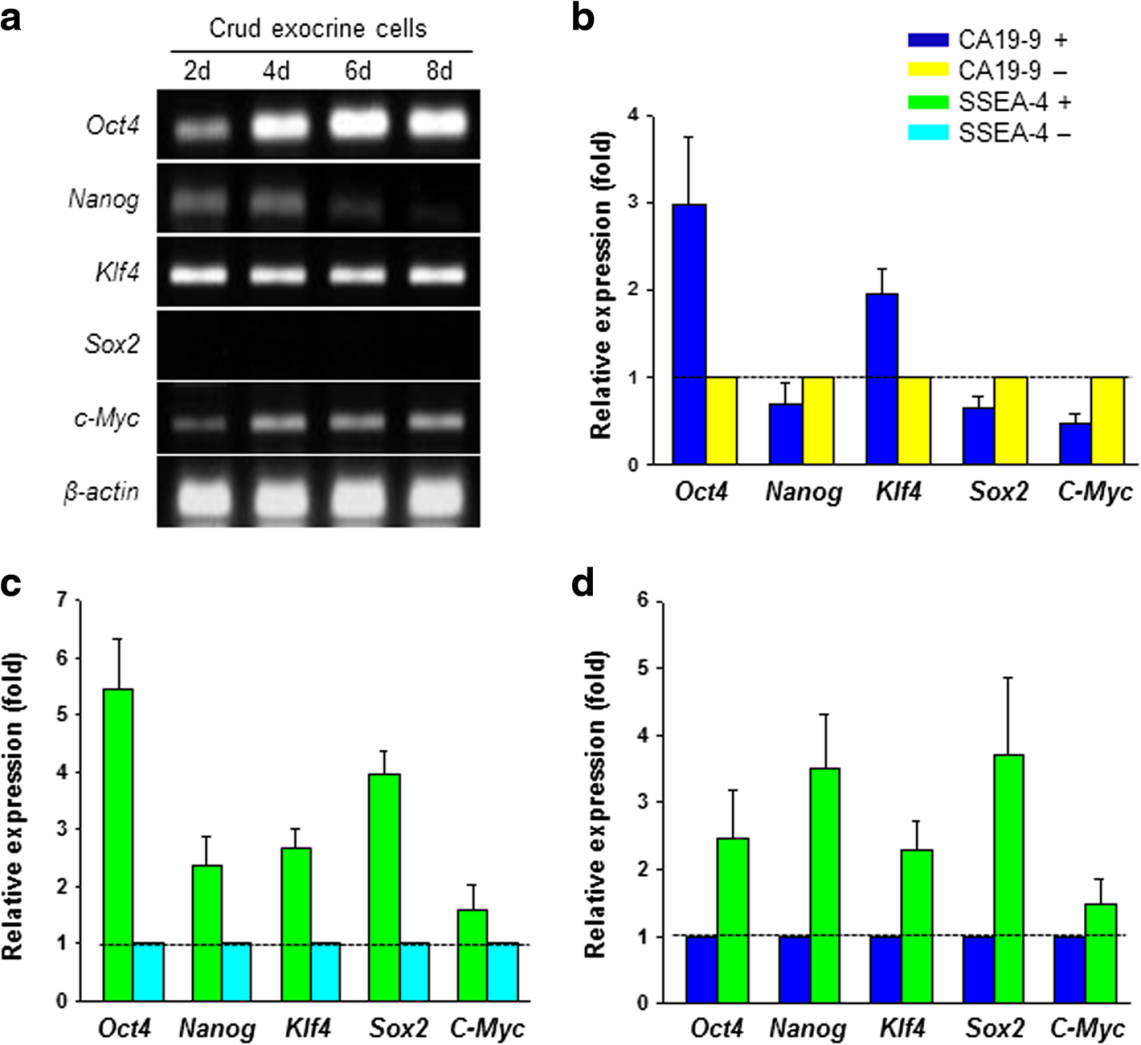
Relative expression of key transcription factors as markers of ES cells. **a** *Oct4*, *Nanog*, *Klf4*, *Sox2*, and *c-Myc* expression in crude exocrine cells on days 2, 4, 6, and 8 of culture by conventional PCR. **b** Expression levels of *Oct4*, *Nanog*, *Klf4*, *Sox2*, and *c-Myc* in CA19-9^+^ and CA19-9^–^ cells by qPCR. **c** Relative expression of *Oct4*, *Nanog*, *Klf4*, *Sox2*, and *c-Myc* in SSEA-4^+^ and SSEA-4^–^ cells by qPCR. **d** Expression of *Oct4*, *Nanog*, *Klf4*, *Sox2*, and *c-Myc* in SSEA-4^+^ and CA19-9^+^ cells by qPCR. *d* days, *SSEA-4* stage-specific embryonic antigen 4

We then extracted RNA from separated pure single-cell suspensions for further characterization of the ES cells. In crude exocrine cells before separation, ES cell-specific markers, such as *Oct4*, *Nanog*, *Klf4*, and *c-Myc*, were found to be expressed in all cultures during the first 8 days of culture; however, Sox2 was not detected by conventional PCR (Fig. 4a). We compared the relative expression levels of ES cell markers between each of the separated cell fractions (CA19-9^+/–^ and SSEA-4^+/–^) by qPCR. CA19-9^+^ cells showed high relative expression levels of *Oct4* and *Klf4*, but low expression of *Nanog*, *Sox2*, and *c-Myc* compared with CA19-9^–^ cells (Fig. 4b). However, the relative expression levels of *Oct4*, *Nanog*, *Klf4*, and *Sox2* were higher in SSEA-4^+^ cells than in SSEA-4^–^ cells (Fig. 4c). In particular, SSEA-4^+^ cells showed high relative expression levels of *Oct4*, *Nanog*, *Klf4*, *Sox2*, and *c-Myc* compared with CA19-9^+^ cells (Fig. 4d). These results showed that although the SSEA-4^+^ cells were present in a portion of pancreatic duct cells, pure separated SSEA-4^+^ cells exhibited characteristics similar to those of ES cells, whereas CA19-9^+^ cells did not.

### SSEA-4^+^ cells differentiated in vivo into adult human pancreatic duct cells

**Fig. 5:**
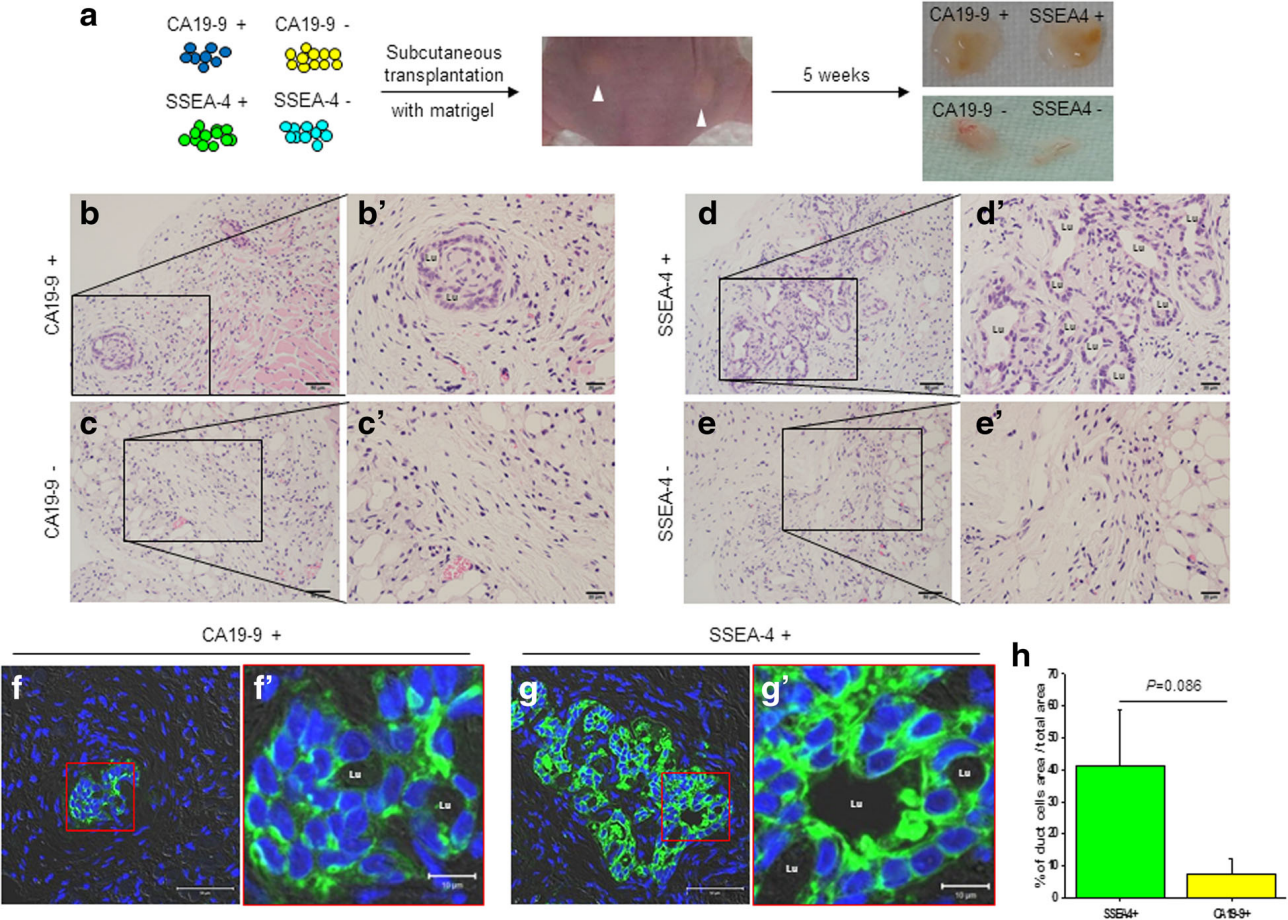
In-vivo differentiation of SSEA-4^+^ cells. **a** Selected pure CA19-9^+^, CA19-9^–^, SSEA-4^+^, or SSEA-4^–^ cells were transplanted into mice and then harvested 5 weeks after transplantation. **b** Morphology of newly formed pancreatic ducts in tissues derived from CA19-9^+^ cells. **b′** Magnified image from **b**. *Lu* lumen. **c** Dermis of the dorsal skin of mice in tissues derived from CA19-9^–^ cells. **c′** Magnified image from **c**. **d** Morphology of newly formed pancreatic ducts in tissues derived from SSEA-4^+^ cells. **d′** Magnified image from **d**. **e** Morphology of tissues derived from mice inoculated with SSEA-4^–^ cells. **e′** Magnified image from **e**. **f** CA19-9 staining in tissue of CA19-9^+^ cells. **f′** Magnified image from **f**. CA19-9-expressing cells in small undifferentiated-like pancreatic ductal cells. **g** CA19-9 staining in tissues of SSEA-4^+^ cells. **g′** CA19-9-expressing cells in actively differentiating pancreatic ductal cells. **h** Area of newly formed pancreatic ductal cells in total tissue area for SSEA-4^+^ cells and CA19-9^+^ cells. *Scale bars*: **b**–**g**, 50 μm; **b′**–**e′**, 20 μm; **f′** and **g′**, 10 μm. *SSEA-4* stage-specific embryonic antigen 4

In this experiment, we could not identify any specific methods for long-term in-vitro culture of pure separated SSEA-4^+^ cells. Therefore, we transplanted pure separated cells into the dorsal subcutaneous region of mice in order to compare the in-vivo ability of the two cell types to differentiate (Fig. 5a). The transplanted pancreatic duct cells (CA19-9^+^ cells) showed few organized pancreatic intralobular ducts (PIDs; Fig. 5b), which consisted of a lumen and cuboidal epithelium surrounding the lumen (Fig. 5b′). However, no epithelial-like cells were detected in the area of the transplanted CA19-9^–^ cells (Fig. 5c), and we could not identify the transplanted cells in the dermis area of the mouse skin (Fig. 5c′). Interestingly, a number of PIDs were observed in transplanted areas of SSEA-4^+^ cells (Fig. 5d). Lumens of various dimensions and cuboidal epithelium were observed in PIDs formed from SSEA-4^+^ cells in vivo (Fig. 5d′). In transplanted SSEA-4^–^ cells, no histological changes were observed, similar to the results of CA19-9^–^ cells (Fig. 5e, e′). Next, we confirmed by immunofluorescence staining whether these newly formed PIDs were pancreatic ducts. In transplanted CA19-9^+^ cells, although few PIDs were formed, CA19-9 was expressed throughout the PIDs (Fig. 5f, f′). Similarly, CA19-9 expression was detected in all newly formed PIDs from SSEA-4^+^ cells (Fig. 5g, g′). The areas of CA19-9-expressing pancreatic duct cells relative to the total transplanted area were 41.02 ± 17.55 % in SSEA-4^+^ cells and 7.50 ± 4.70 % in CA19-9^+^ cells (Fig. 5h).

### In-vivo differentiated pancreatic duct cells derived from SSEA-4^+^ cells expressed Sox9 and Ngn3

**Fig. 6:**
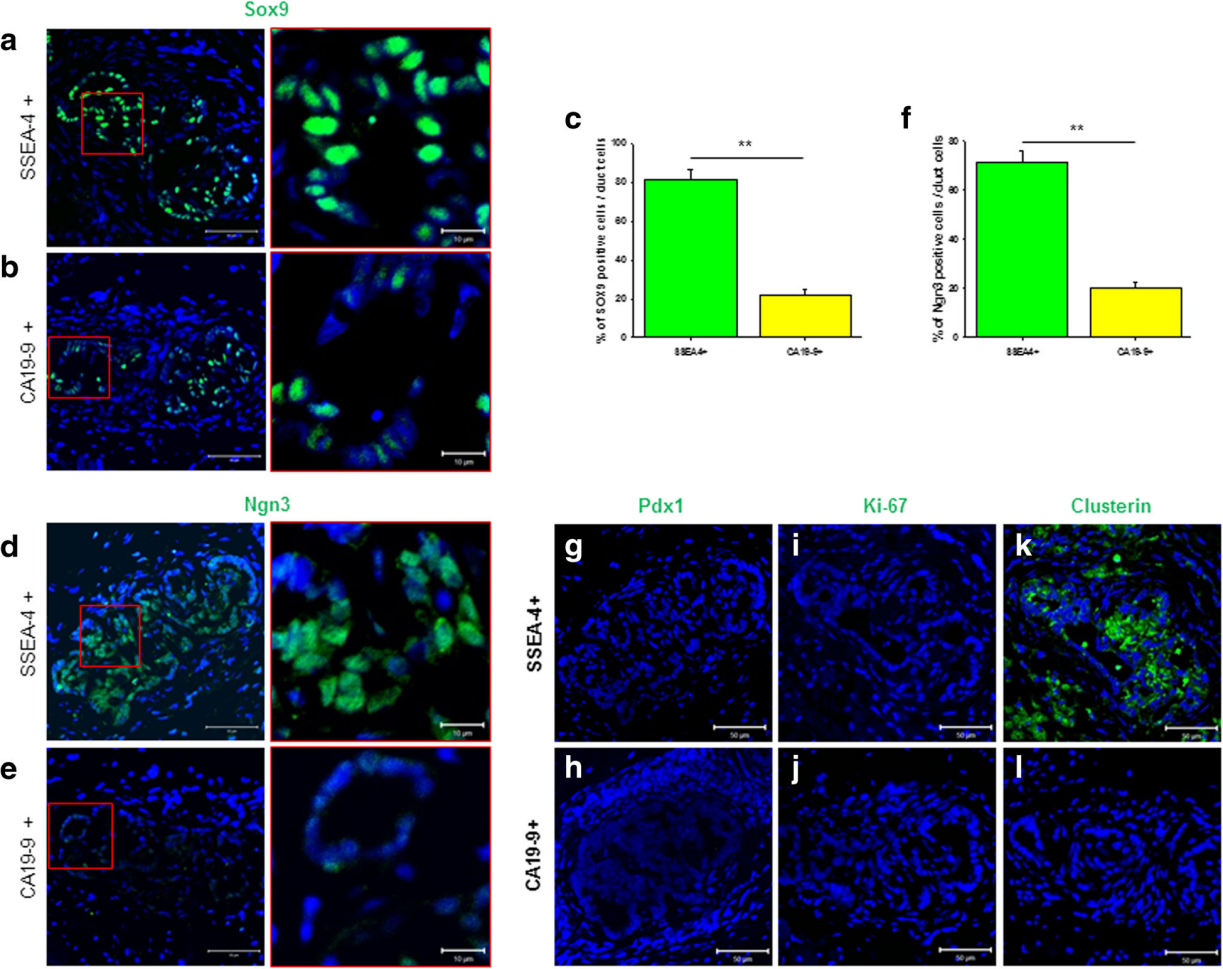
Expression of Sox9 and Ngn3 in newly formed pancreatic ductal cells. **a** Sox9 expression in the nuclei of newly formed pancreas ductal cells from tissues derived from SSEA-4^+^ cells. *Scale bars*: *left panel*, 50 μm; *right panel*, 10 μm. **b** Sox9 expression in newly formed pancreas ductal cells from tissues derived from CA19-9^+^ cells. *Scale bars*: *left panel*, 50 μm; *right panel*, 10 μm. **c** Percentage of Sox9^+^ cells in total pancreatic ductal cells from tissues derived from SSEA-4^+^ or CA19-9^+^ cells. **P = 0.0005. **d** Ngn3 expression in tissues derived from SSEA-4^+^ cells. *Scale bars*: *left panel*, 50 μm; *right panel*, 10 μm. **e** Ngn3 expression in tissues derived from CA19-9^+^ cells. *Scale bars*: *left panel*, 50 μm; *right panel*, 10 μm. **f** Percentage of Ngn3^+^ cells in total pancreatic ductal cells in tissues derived from SSEA-4^+^ or CA19-9^+^ cells. **g** Pdx1 expression in tissues derived from SSEA-4^+^ cells. **h** Pdx1 expression in tissues derived from CA19-9^+^ cells. **i** Ki-67 expression in newly formed pancreatic ductal cells from tissues derived from SSEA-4^+^ cells. **j** Ki-67 expression in newly formed pancreatic ductal cells from tissues derived from CA19-9^+^ cells. **k** Clusterin expression in newly formed pancreatic ductal cells from tissues derived from SSEA-4^+^ cells. **l** Clusterin expression in newly formed pancreatic ductal cells from tissues derived from CA19-9^+^ cells. *Scale bars*: **g**–**l**, 50 μm. *SSEA-4* stage-specific embryonic antigen 4

To identify the specific genes associated with pancreas differentiation, newly formed PIDs from SSEA-4^+^ cells, CA19-9^+^ cells, or normal adult human pancreatic ducts were evaluated using immunofluorescence staining. Human adult pancreatic ducts, which consisted of pancreatic main ducts (PMDs) and PIDs, excluding acinar cells and islets, were isolated from pancreatic tissue in order to analyze control cell morphology and gene expression profiles (Additional file 2: Figure S1a). Pancreatic duct cells and morphology were detected without acinar cells and islets in H&E slide sections of both PMDs and PIDs (Additional file 2: Figure S1b, c). Many small duct cells, including the lumen, were detected in PMD tissues, similar to those in PIDs from SSEA-4^+^ cells (Additional file 2: Figure S1b′); however, long extended ducts were detected in PID tissues (Additional file 2: Figure S1c′). To identify specific gene expression patterns associated with pancreas differentiation in pancreatic duct cells, the expression of the major transcription factor Sox9, which is associated with pancreatic development, and Ngn3, a key transcription factor involved in endocrine cell development, was confirmed by immunofluorescence staining. Sox9 expression was specifically detected in the nuclei of PIDs from SSEA-4^+^ cells (Fig. 6a) and normal pancreatic duct tissue (Additional file 2: Figure S1d) but was weakly expressed in PIDs from CA19-9^+^ cells (Fig. 6b).

The percentage of Sox9-expressing cells in total PIDs was considerably higher in PIDs from SSEA-4^+^ cells than in PIDs from CA19-9^+^ cells (81.54 ± 5.22 % versus 21.96 ± 2.7 %, respectively; Fig. 6c). The expression of Ngn3 in nuclei of PIDs was detected in PIDs from SSEA-4^+^ cells and PIDs from CA19-9^+^ cells; however, staining was observed in nearly all PIDs from SSEA-4^+^ cells (Fig. 6d) but only in few PIDs from CA19-9^+^ cells (Fig. 6e). Ngn3 expression was also highly detected in normal pancreatic duct tissue (Additional file 2: Figure S1e). The percentage of cells expressing Ngn3 of the total PID cells was higher in PIDs from SSEA-4^+^ cells than in PIDs from CA19-9^+^ cells (70.99 ± 5.17 % versus 19.98 ± 2.54 %; Fig. 6f). However, Pdx1, a major transcription factor involved in pancreatic development, was not detected in PIDs from SSEA-4^+^ cells (Fig. 6g) or in PIDs from CA19-9^+^ cells (Fig. 6h). Similarly, Pdx1 expression was not observed in adult pancreatic duct tissue (Additional file 2: Figure S1f). We confirmed proliferation of newly formed PIDs by immunostaining with Ki-67, a proliferation marker, to determine whether these cells exhibited proliferative activity similar to ES cells; however, Ki-67-expressing cells were not detected in PIDs from SSEA-4^+^ cells (Fig. 6i) or in PIDs from CA19-9^+^ cells (Fig. 6j), and few Ki-67 cells were detected in adult PID tissues (Additional file 2: Figure S1g). Finally, we confirmed the expression of clusterin, a pancreatic differentiation marker, in PIDs and detected high expression of this marker in peripheral PIDs derived from SSEA-4^+^ cells (Fig. 6k, l).

## Discussion

In this study, we evaluated the characteristics and features of ES-like cells expressing SSEA-4 in the adult human pancreas. To confirm the characteristics of cells expressing SSEA-4, we compared the cells with cells costained with Ck19 or amylase. Immunostaining showed that CA19-9^+^ cells were detected in around cell clusters, and that amylase-positive cells were present within the cell clusters. SSEA-4^+^ cells were found at the edges of cell clusters. Although some cells coexpressed SSEA-4 and CA19-9 or amylase, these findings were not reliable for quantitative analysis (Additional file 3: Figure S2b–g). However, FACS analysis showed that 9.65 ± 2.68 % of normal-sized cells coexpressed SSEA-4 and CA19-9, whereas 7.72 ± 0.86 % of small-sized cells coexpressed these targets. In addition, CK19 was specifically expressed in CA19-9^+^ cells, whereas amylase was not; amylase was only expressed in the CA19-9-negative population (Additional file 3: Figure S2a). Although weak CK19 expression was detected in CA19-9-negative cells, this expression was thought to be due to the terminal duct cells connected with exocrine cells directly. Overall, the expression of SSEA-4 was greater in CA19-9^+^ cells than CA19-9^–^ cells. Although SSEA-4 expression has been found in dividing CA19-9^+^ and CA19-9^–^ cells, the cells expressing SSEA-4 seemed to be distributed in the duct cells. However, when using the anti-SSEA-4 antibody to separate the cells, only cells expressing SSEA-4 were more effectively purified than when using anti-CA19-9 antibodies. These results were intriguing because almost all SSEA-4^+^ cells were present in pancreatic ducts, which are known to contain stem/progenitor cells and multipotent stem cells [17, 18]. Notably, the distribution of SSEA-4^+^ cells was not associated with cell size.

Moreover, SSEA-4^+^ cells expressed ES cell-specific transcription factors, with higher expression levels than those in pancreatic duct cells expressing CA19-9, suggesting that SSEA-4^+^ cells in the adult human pancreas may exhibit gene expression patterns similar to those of ES cells. However, SSEA-4^+^ cells did not exhibit rapid proliferation rates and self-renewal ability, and the viability of SSEA-4^+^ cells was gradually decreased in vitro under single-cell culture conditions. Finally, in-vitro passage culture of epithelial-like primary pancreas exocrine cells was not possible; however, mesenchymal-like stem cells contained in the crude exocrine cells were able to be passaged continually (data not shown). Our data provided important insights into the characteristics of SSEA-4^+^ cells in pancreatic ducts.

Although we have not determined specific in-vitro culture conditions for continuous growth of SSEA-4^+^ single cells, we did show that isolated SSEA-4^+^ single cells could differentiate into PIDs in vivo after transplantation into the dorsal subcutaneous region of mice. In this study, we focused on the differentiation capacity and regenerating activity of cells expressing SSEA-4 and found newly formed PID structures within the area of transplanted SSEA-4^+^ cells. Although few newly formed PIDs were detected in areas of transplanted CA19-9^+^ cells, these PIDs formed in smaller areas than those in the group transplanted with SSEA-4^+^ cells and showed lower expression of Sox9 and Ngn3. These results demonstrated that there were few cells expressing SSEA-4^+^ in the transplanted population of CA19-9^+^ cells, and that there were not sufficient numbers of cells to induce differentiation and regeneration. Specifically, it was difficult to determine whether the transplanted CA19-9^+^ cells simply proliferated in vivo because the cell populations, including CA19-9^+^ cells, showed almost no proliferation during both in-vitro culture and after transplantation in vivo.

SSEA-4^–^ and CA19-9^–^ cells also did not differentiate to PIDs in vivo because SSEA-4^+^ cells were not present in these cell populations. Although a small number of cells expressed SSEA-4 presence in CA19-9^–^ cell populations, these cells were mixed with a number of exocrine cells expressing amylase. Therefore, we concluded that SSEA-4^–^ or CA19-9^–^ cells may not differentiate into PIDs due to impurities and the presence of a small number of cells expressing SSEA-4 in vivo.

Newly formed pancreatic ducts are distinctly detected during regeneration of the pancreas after mechanical injury using partial pancreatectomy (pPx) [19, 20] or duct ligation [21, 22] in rodents. Furthermore, these ducts directly differentiate into exocrine cells, such as acinar cells, or into endocrine cells (e.g., insulin-producing cells) [23, 24]. For these reasons, many studies have reported that multipotent stem cells/progenitor cells, which may able to induce the differentiation/regeneration of the pancreas, are present in pancreatic ducts and newly formed ducts [25–27]. In this study, we found that these newly formed pancreatic ducts expressed Sox9, which is involved in progenitor proliferation and differentiation in both developmental systems [28–30] and plays a role in pancreatic development, including islet development [30, 31]. We confirmed the expression of CA19-9 in newly formed PIDs by immunofluorescence staining. All newly formed PIDs in the total transplantation area were stained with CA19-9, and Sox9 and Ngn3 were also detected in the nuclei of cells constituting the newly formed PIDs. Therefore, the percentages of cells expressing Sox9 and Ngn3 were calculated relative to the total number of PIDs. Notably, Sox9 was expressed throughout the PMDs and PIDs, and newly formed PIDs from SSEA-4^+^ cells showed a Sox9 expression pattern in vivo similar to that of the adult pancreatic duct. Thus, these data demonstrated that isolated SSEA-4^+^ cells from the adult human pancreas may be able to differentiate into functional pancreatic ducts expressing Sox9 in vivo, similar to adult pancreatic ducts.

Ngn3, which is associated with the development and differentiation of the pancreas, was also detected in adult pancreatic ducts and newly formed PIDs from SSEA-4^+^ cells. Ngn3 is a marker of endocrine progenitors [32, 33] and neogenesis [34, 35] in the adult pancreas. Additionally, Pdx1-expressing cells were confirmed in newly formed PIDs because Pdx1 is a critical transcription factor in the embryologic development [36, 37] and regeneration of the pancreas [38]. However, Pdx1 was not detected in PIDs derived from SSEA-4^+^ cells or CA19-9^+^ cells or in adult pancreatic ducts.

In our study, no proliferative cells were detected in newly formed PIDs in vivo, regardless of the cell phenotype. These data indicated that although SSEA-4^+^ cells may have gene expression patterns similar to those of ES cells, proliferation activity may be suppressed because the tissues are adult tissues. Clusterin is glycoprotein that induces neogenic ductule and islet formation during regeneration of the pancreas after injury, such as pPX. Clusterin is usually expressed strongly in regenerating ductal cells and developing acinar cells. In this study, clusterin was generally expressed in only newly formed PIDs from SSEA-4^+^ cells. Therefore, although newly formed PIDs were observed in the transplanted areas of CA19-9^+^ cells and SSEA-4^+^ cells, newly formed PIDs all contained SSEA-4^+^ cells, suggesting the occurrence of regeneration and differentiation. In this study, we confirmed that SSEA-4^+^ cells may differentiate into pancreatic ducts during the early developmental stage of pancreas formation, and we expected that SSEA-4^+^ cells may differentiate into endocrine cells, including insulin-producing β cells. However, insulin-expressing cells were not detected in PIDs derived from either cell type (data not shown). This may be because the transplantation area did not have appropriate environmental factors for differentiation into endocrine cells. Therefore, further studies are needed to achieve transplantation into the appropriate location, such as the pancreas, in order to observed differentiation of SSEA-4^+^ cells into β cells.

## Conclusions

Our data indicated that SSEA-4^+^ cells in the adult human pancreas may exhibit transcription factor expression patterns similar to those of ES cells and therefore were able to differentiate in vivo into developing pancreatic duct cells expressing Sox9 and Ngn3. These data suggested that isolated SSEA-4^+^ cells derived from the adult human pancreas may have applications as pancreatic stem/progenitor cells for differentiation and regeneration of the adult pancreas. In addition, these SSEA-4^+^ cells may have practical uses in the differentiation/regeneration of insulin-producing cells. Further studies are needed to investigate these possibilities.

## Additional files

Additional file 1 is Table S1 presenting PCR primer sequences. a Gene-specific primers for reverse-transcription-PCR. b Gene-specific primers for qPCR. (TIF 89 kb)
Additional file 2 is Figure S1 showing morphology of adult human pancreatic ducts. a Adult human pancreatic ducts were isolated from partial pancreatic tissue using a dissecting microscope. Tissues were divided into PMDs and PIDs. b Morphologies of PMDs. b′ Magnified image from b. c Long outstretched duct structures in PIDs. c′ Magnified image from c. d Sox9-positive cells in the nucleolus of pancreatic ductal cells. e Ngn3 expression in ductal cells. f Pdx1 was not detected in adult pancreatic ductal cells. g Ki-67 staining for detection of proliferation in pancreatic ductal cells. (TIF 852 kb)
Additional file 3 is Figure S2 showing identification of cells expressing SSEA-4. a Identity of separated cell populations was confirmed by PCR. Cells expressing SSEA-4 were detected in both CA19-9^+^ cell populations and SSEA-4^+^ cell populations. However, cytokeratin 19 and amylase were detected in SSEA-4^+^ cell populations. b SSEA-4 staining was detected in exocrine cells. c Cytokeratin 19 was stained in exocrine cells. d Merged image of SSEA-4 staining and CK19 staining. *Arrows* in b–d indicate SSEA-4 and CK19 coexpressing cells. e SSEA-4 staining was detected in exocrine cells. f Amylase-expressing exocrine cells. g Merged image of SSEA-4 staining and amylase staining. *Arrows* in e–g indicate SSEA-4 and amylase coexpressing cells. (TIF 647 kb)

## Abbreviations

DAPI: 4′,6-Diamidino-2-phenylindole
DMEM: Dulbecco’s modified Eagle medium
ES: Embryonic stem
FAM: 6-Carboxyfluorescein
FBS: Fetal bovine serum
H&E: Hematoxylin and eosin
MACS: Magnetic-activated cell sorting
Ngn3: Neurogenin3
PBS: Phosphate-buffered saline
PE: IgG-phycoerythrin, FACS, Fluorescence-activated cell sorting
PID: Pancreatic intralobular duct
PMD: Pancreatic main duct
pPx: Partial pancreatectomy
qPCR: Quantitative polymerase chain reaction
SSEA-4: Stage-specific embryonic antigen 4
TAMRA: Carboxytetramethylrhodamine
VSEL: Very small embryonic-like stem cell
