# Supplementary figures and images for: Adult human pancreas-derived cells expressing stage-specific embryonic antigen 4 differentiate into Sox9-expressing and Ngn3-expressing pancreatic ducts in vivo
Source: Stem Cell Res Ther. 2016 Nov 11;7:162. doi: 10.1186/s13287-016-0422-0 (PMC5105312; doi:10.1186/s13287-016-0422-0)

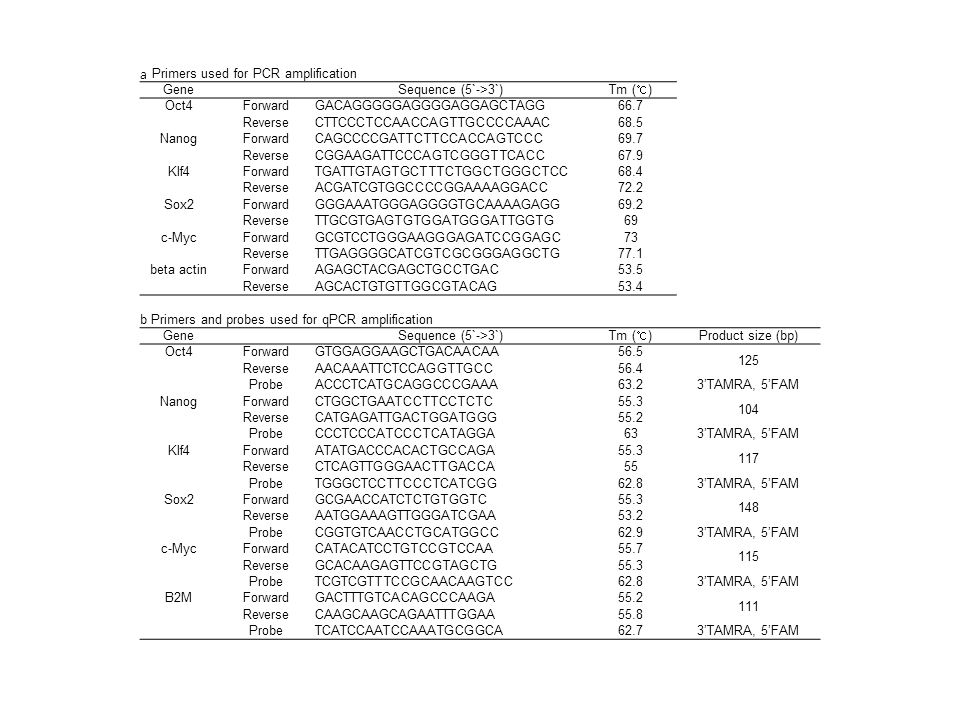

Supplement: Additional file 1 — is Table S1 presenting PCR primer sequences. a Gene-specific primers for reverse-transcription-PCR. b Gene-specific primers for qPCR. (TIF 89 kb) [file 13287_2016_422_MOESM1_ESM.tif]

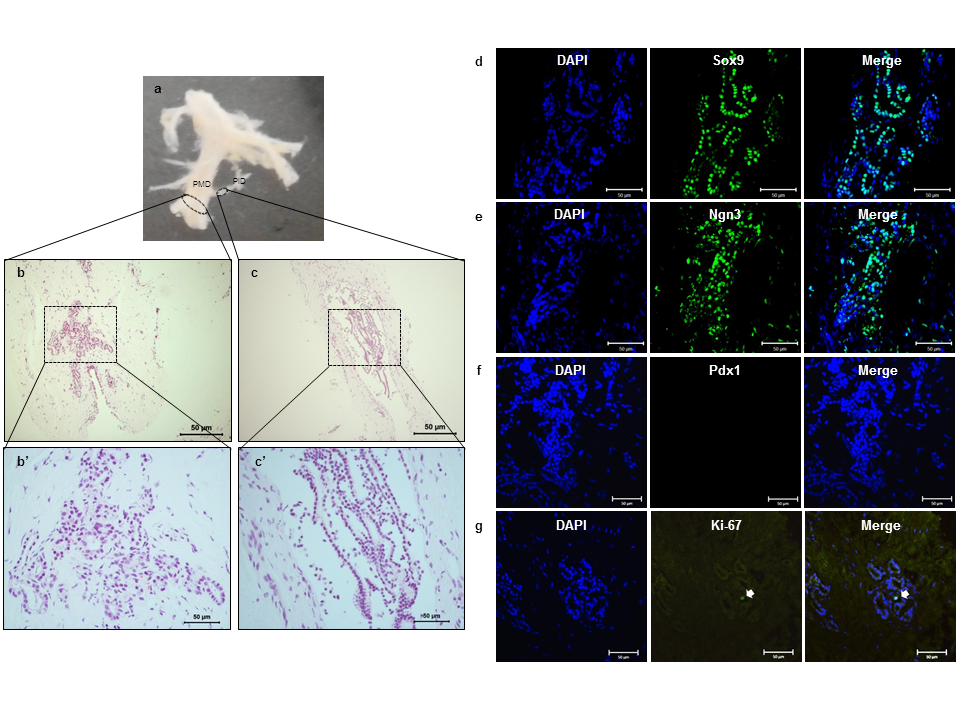

Supplement: Additional file 2 — is Figure S1 showing morphology of adult human pancreatic ducts. a Adult human pancreatic ducts were isolated from partial pancreatic tissue using a dissecting microscope. Tissues were divided into PMDs and PIDs. b Morphologies of PMDs. b′ Magnified image from b. c Long outstretched duct structures in PIDs. c′ Magnified image from c. d Sox9-positive cells in the nucleolus of pancreatic ductal cells. e Ngn3 expression in ductal cells. f Pdx1 was not detected in adult pancreatic ductal cells. g Ki-67 staining for detection of proliferation in pancreatic ductal cells. (TIF 852 kb) [file 13287_2016_422_MOESM2_ESM.tif]

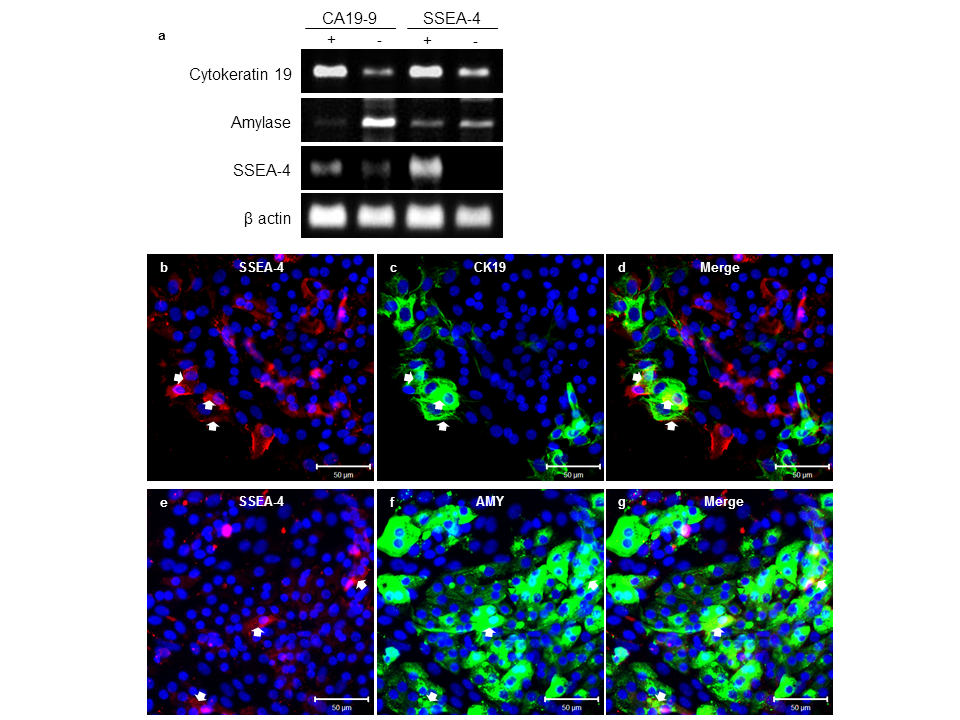

Supplement: Additional file 3 — is Figure S2 showing identification of cells expressing SSEA-4. a Identity of separated cell populations was confirmed by PCR. Cells expressing SSEA-4 were detected in both CA19-9+ cell populations and SSEA-4+ cell populations. However, cytokeratin 19 and amylase were detected in SSEA-4+ cell populations. b SSEA-4 staining was detected in exocrine cells. c Cytokeratin 19 was stained in exocrine cells. d Merged image of SSEA-4 staining and CK19 staining. Arrows in b–d indicate SSEA-4 and CK19 coexpressing cells. e SSEA-4 staining was detected in exocrine cells. f Amylase-expressing exocrine cells. g Merged image of SSEA-4 staining and amylase staining. Arrows in e–g indicate SSEA-4 and amylase coexpressing cells. (TIF 647 kb) [file 13287_2016_422_MOESM3_ESM.tif]
